# Tau Pathology in Parkinson's Disease

**DOI:** 10.3389/fneur.2018.00809

**Published:** 2018-10-02

**Authors:** Xue Zhang, Fei Gao, Dongdong Wang, Chao Li, Yi Fu, Wei He, Jianmin Zhang

**Affiliations:** Department of Immunology, Research Center on Pediatric Development and Diseases, Institute of Basic Medical Sciences, Chinese Academy of Medical Sciences and School of Basic Medicine, Peking Union Medical College, State Key Laboratory of Medical Molecular Biology, Beijing, China

**Keywords:** tauopathy, Parkinson's disease (PD), hyperphosphorylation, alpha-synuclien, tau protein

## Abstract

Tau protein—a member of the microtubule-associated protein family—is a key protein involved in many neurodegenerative diseases. Tau pathology in neurodegenerative diseases is characterized by pathological tau aggregation in neurofibrillary tangles (NFTs). Diseases with this typical pathological feature are called tauopathies. Parkinson's disease (PD) was not initially considered to be a typical tauopathy. However, recent studies have demonstrated increasing evidence of tau pathology in PD. A genome-wide association (GWA) study indicated a potential association between tauopathy and sporadic PD. The aggregation and deposition of tau were also observed in ~50% of PD brains, and it seems to be transported from neuron to neuron. The aggregation of NFTs, the abnormal hyperphosphorylation of tau protein, and the interaction between tau and alpha-synuclein may all contribute to the cell death and poor axonal transport observed in PD and Parkinsonism.

## Introduction

Tau protein is produced from a single human gene named microtubule-associated protein tau (MAPT), which is located on chromosome 17 and encodes a cytoskeletal protein that stabilizes microtubules (1). Although tau is widely distributed in neurons of the central nervous system (CNS), its levels in CNS astrocytes and oligodendrocytes are low (2). Tau proteins play a role in stabilizing microtubules, binding to membrane, and regulating axonal transport (3–5). Under physiological conditions, tau is highly soluble and unfolded. However, with changes in isoforms or phosphorylation patterns in pathological states, tau proteins become insoluble and misfolded, causing damage to neurons and axonal transport (6, 7). Protein misfolding, accumulation, and aggregation have been observed in many neurodegenerative diseases (8), which may contribute to neuron damage and neurological disorders. The pathological aggregation of tau or neurofibrillary tangles are known as tauopathy, an important hallmark of many human neurodegenerative disease, such as Alzheimer's disease (AD) and Parkinson's disease (PD) (9, 10).

Parkinson's disease, named after Dr. James Parkinson, is a major neurodegenerative disease that primarily affects motor systems but can also be accompanied by cognitive and behavioral problems (11). There is a widespread neuron degeneration in PD brains, affecting up to 70% of dopaminergic neurons in the substantia nigra (SN) by the time of death (12, 13). The neuropathological hallmarks of PD include Lewy bodies (LBs) in the SN, brainstem, and rostral and forebrain regions and the selective deletion of dopaminergic neurons in the SN (14, 15). Cell-death induced damage in SN may be the source of patient movement disorders. Although the causes of this cell death are generally unclear, researchers have observed an enrichment of tau protein and alpha-synuclein in neuronal Lewy bodies, which may be related to tauopathy in PD (16). Immunohistochemistry with anti-tau antibodies showed high level of NFTs in the substantia nigra from post-mortem human brain tissue (17). Researchers have also reported that tauopathies in PD and PD with dementia (PDD) were only observed in DA neurons of the nigrostriatal region, which contrasts with the wide-spread expression pattern of tau throughout the entire brain in AD (18).

**Figure 1:**
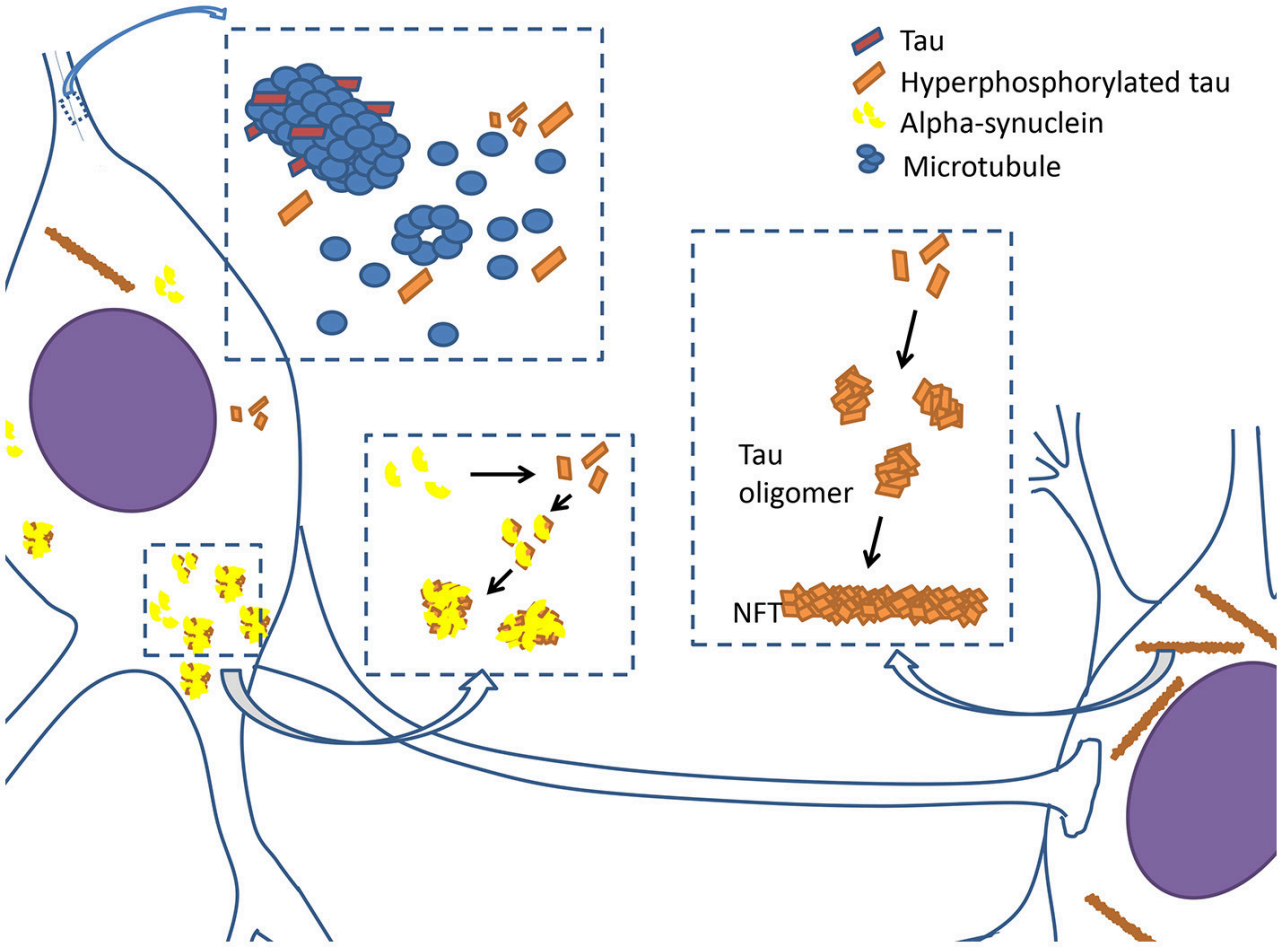
Tauopathy in PD. Tau proteins are integrated with microtubules and stabilize microtubules in physiological condition. However, when tau proteins are hyperphosphorylated, they begin to disintegrate from microtubules, causing neuronal dysfunction. Hyperphosphorylated tau proteins prone to assemble together to form oligomers and develop into filamentous neurofibrillary tangles (NFTs). Hyperphosphorylated tau proteins also interact with α-synuclein to promote aggregation and fibrilization each other, and subsequently cause the formation of lewy bodies and axonal transport dysfunction.

Although tau pathology in AD and other tau-associated neurodegenerative diseases have been previously described, the importance of tau pathology in PD has been undervalued. Therefore, we reviewed the tau pathologies that might be involved in PD (Figure 1), seeking to identify tau as a potential therapeutic target.

## Assembled Tau in PD

### Structure of normal TAU

There are six different isoforms of tau protein in the human brain, with the differences among them resulting from alternative mRNA splicing of a single gene located on chromosome 17 (19). The microtubule-binding domains of the protein consist of adjoining sequence and repeat sequences. The six isoforms are divided into two categories based on the number of these repeats, namely, 3R and 4R. The 3R tau isoform has three repeats, while 4R tau has four repeats (20). Each of the repeats is able to bind to microtubules, and the more repeats the protein has, the stronger affinity it will have with them (21). Therefore, when compared with 3R tau, 4R tau is considerably easier to bundle with and polymerize microtubules. In different tauopathies, the pathological tau protein has different isoforms and conformations. Progressive supranuclear palsy (PSP) and corticobasal degeneration (CBD) are both associated with Parkinson's disease and are associated with 4R tau deposits in neurons and microglia (22). A study of a multigenerational family suffering from X-linked parkinsonian syndrome also showed a strong 4R tauopathy in the striatum (23).

### Assembled TAU in PD and other neurodegenerative diseases

Tau protein is soluble and unfolded under physiological conditions; however, in many neurodegenerative diseases, tau appears to be insoluble and assembled (24). The most probable mechanism of tau assembly involves mutations of the microtubule-associated protein tau (MAPT). MAPT is a single gene located on chromosome 17q21, containing 16 exons (25). A genome-wide association (GWA) study for sporadic PD cases in Europe confirmed that MAPT is closely linked to sporadic PD (26). MAPT is divided into two haplogroups called H1 and H2 based on whether the gene is in the inverted orientation (27). A meta-analysis in Caucasian populations reported that the H2 haplotype is more relevant to PD than the H1 haplotype, as the risk of suffering from PD is lower in Caucasians with the H2 haplotype (28). Certain FTDP-17 mutations, including missense mutations, deletions in the coding region, and intronic mutations, result in tau aggregation. This aggregation can cause dominantly inherited frontotemporal dementia and Parkinsonism linked to chromosome 17 (29). Most missense mutations in the coding region tend to affect how well tau can associate with microtubules (30). However, studies also show that some missense mutations such as G272V, P301L, and P301S may play an important role in filament assembly because they markedly facilitated the propensity for tau to assemble (31). If the mutation is in the repeat region or if changes in the relative amounts of 3R tau or 4R tau could lead to overproduction of 4R tau, the filament morphology tends to be incorrectly over-folded (32). Tau deposition and assembled filaments are observed in many neurodegenerative diseases and are considered a typical neuropathological hallmark. Significantly lower levels of soluble tau and a lower 3R-tau to 4R-tau ratio has been shown in the SN of patients with PD (33), indicating tauopathy similarity between PD and AD. PSP and CBD are subtypes of Parkinson's disease known as Parkinson-plus syndromes, and both are associated with the formation of tau deposits. Filamentous tau deposits can be observed in neurons and microglia in these diseases (34).

### Prion-like pathological TAU spreading in animal models and patients with PD

Increasing evidence shows that tau aggregation and deposition contribute to PD pathology. Thus, to best understand the mechanisms underlying PD pathogenesis, early diagnosis, and treatment, determining how tau aggregation spreads to other areas is imperative. Researchers have observed Lewy bodies in grafted neurons that patients with PD received as transplants (35). The assumption was that neurofibrillary lesions spread along the neuronal pathways in the brain. Recent evidence shows that misfolded tau can move from cell to cell, similar to prion disease (36). Clavaguera and other researchers inserted a mutant human tau transgene into mice to show that human tau can be transported from neuron to neuron (37). Another study has shown that the spreading of the tau inclusions depended on the initial injection site of synthetic tau fibrils. The pathological tau were more likely to spread through functionally connected neuroanatomical pathways rather than through adjacent anatomical locations (38).

After showing prion-like transmission and spreading of tauopathy by injecting pathological tau from the human brain into transgenic ALZ17 mice, researchers assessed the role that different tau strains play in this pathological process. By separately injecting human brain homogenates from patients who suffered from argyrophilic grain disease (AGD), PSP, and CBD into different ALZ17 mice, researchers demonstrated that the different tau isoforms may induce different tauopathies. Mice receiving CBD or AGD tau differentially displayed silver-positive or silver-negative astrocytic plaques that matched the injection patterns and that were highly similar to the types of tau-related pathological damage typically found in the brains of patients suffering from the respective diseases (39). Furthermore, 12 months after injecting ALZ17 mice with brain homogenate from mice that had been injected with the human tau P301S transgene 18 months earlier, the ALZ17 mice showed fewer tau inclusions than those of mice that had been injected with AGD brain extracts (40). Similar phenomena were observed in mice injected with filament 4R-tau strains formed in HEK293T cells (41). Further, in patients with PD who received cell-replacement therapy to repair brain damage, hyperphosphorylated tau such as phospho-tau Ser202 and Thr205 were found in grafted neurons years after transplantation (42). Taken together, these findings demonstrate tau strain-specific prion-like transmission and spreading in the disease state, including in PD. Additionally, the specific strain plays an important role in causing distinct pathologies.

### Abnormal hyperphosphorylation of the TAU protein

Hyperphosphorylation of the tau protein is another mechanism through which tau might accumulate and form filaments, which might also influence the ability of tau to bind to microtubules, possibly limiting how microtubules can be combined and resulting in their aggregation into NFTs (43). In this way, the microtubules might disintegrate, eventually leading to the impaired transport capacity of axon microtubules. Tau protein appears to be easily phosphorylated because of its 85 potential phosphorylation sites, and researchers have characterized over 20 kinases that may be related to the phosphorylation of tau protein after its transcription (44). With respect to the healthy human brain, there are only two or three phosphorylated amino acid residues in tau protein, while there may be considerably more in brains exhibiting tauopathy (45). Additional research indicates that the most likely mechanism underlying the hyperphosphorylation is either upregulated protein-kinase activity or downregulated protein-phosphatase activity (46). Among protein kinases, GSK-3β (a proline-directed protein kinases) and CDK5 (a non-PDPK non-proline-directed protein kinase) are probably the two most important kinases in tauopathy. Using neuronal stem/progenitor cells and transgenic mice, researchers have demonstrated a pivotal role for GSK-3β in the interaction between DA neurons and astrocytes during damage and recovery (47), which might be related to the death of DA neurons in PD. Further, the application of CDK5 in cortex suffering from Lewy body disease was reported in 2000, indicating that CDK5 may participate in the formation of Lewy bodies (48). In contrast, unlike GSK-3β and CDK5, a series of protein phosphatases (PP-2A, PP-2B, and PP-1) can dephosphorylate protein tau *in vitro* and *in vivo*, which may act to protect against tauopathy (49). Reduced activity levels of PP-2A in the brains of patients with PD and AD indirectly confirmed this inference (50, 51).

Hyperphosphorylation of tau protein is an important step in tau aggregation and the formation of neurofibrillary tangles (52). Antibodies targeting p-taus were able to detect tau isoforms in brain tissue suffering from sporadic PD or dementia with Lewy bodies, indicating the existence of hyperphosphorylated tau protein in NFTs (53). Tau aggregation more easily begins from the C-terminus of the protein (54). Therefore, kinases that phosphorylate at the C-terminus might be crucial for the formation of tau filaments and aggregates. For example, an *in vitro* study indicated that the GSK-3β associated with the phosphorylation of tau at the C-terminal had an ability to promote the fibrillation of the protein, while the level of microtubule assembly stayed low due to DYRK1A (55). A study on the structure and dynamics of phosphorylated tau filaments using computer simulations indicated that the all the masses and charges had changed because of phosphorylation at regions associated with microtubules, resulting in further aggregation of tau (56).

Phosphorylated tau is also proved to be related to the N-methyl-D-aspartic receptor (NMDAR) at postsynaptic sites (57), which suggests that the toxic pathology of tau phosphorylation is associated with the synapse. Meanwhile, the FTDP-17 tau mutant, which is known to be associated with PDD, was shown to interfere with synaptic vesicles in presynaptic terminals, causing the dysfunction of vesicle traffic and presynaptic activity (58). There are also a number of studies showing that the hyperphosphorylation of tau protein may depolymerize microtubules, causing their dysfunction, impaired axonal transport, and ultimately cell death (59). Okadaic acid, an inhibitor of tau phosphorylation, was used to investigate the synaptic structure of neurons in rats (60). This study highlights the potential relationship between phosphorylated tau and the loss of synaptic function. Similar results have been shown in animal models. Transgenic mice expressing human tau P301L show Parkinsonism as early as 6.5 months (61), while a similar phenotype occurs in the K3 mouse model that expresses human tau with the K396I mutation. In this latter case, Parkinsonism symptoms can be improved using L-dopa (62).

### P-TAU associated with alpha-synuclein leads to toxic injury in PD

One of the key proteins involved in PD pathology is alpha-synuclein, a highly soluble neuronal cytoplasmic protein that is localized to presynaptic elements in the CNS (63). Under certain conditions, such as missense mutations, post-translational modifications (e.g., phosphorylation and C-terminal truncation), and peroxynitrite stimulation, alpha-synuclein is prone to being fibrillated and to residing in Lewy bodies with other proteins (64), which is a feature of PD that occurs along with Lewy neurites.

Researchers found that tau protein, especially phosphorylated tau, existed in Lewy bodies along with alpha-synuclein and that neurofibrillary tangles could be observed around Lewy bodies (53, 65, 66). This phenomenon led researchers to hypothesize a positive interaction between tau and alpha-synuclein. A transgenic mouse model of PD showed increased levels of p-tau and the co-localization and overexpression of alpha-synuclein and p-tau, which were deposited in large inclusion bodies that are considered similar to Lewy bodies in PD (67). A series of experiments *in vitro* indicate that tau incubated with synthesized alpha-synuclein oligomers can induce all forms of tau, including the assembly of toxic tau forms (68). Furthermore, studies using QBI293 cells demonstrated that alpha-synuclein induces tangles of tau and promotes phosphorylation of tau in cells (69). Reports also indicate that the nucleus of neurons were surrounded by alpha-synuclein and human tau with the P301L mutation, which may be to blame for the loss of neuronal function (70). Researchers have been able to successfully detect the aggregation of the two proteins in brains of patients with PD using two novel antibodies specific to oligomeric tau and alpha-synuclein (71). Similar results were shown in a transgenic mouse model that was inoculated with alpha-synuclein supplied from preformed fibrils, indicating that the existence of both alpha-synuclein and tau promotes fibrillation, and this phenomenon is also confirmed in human brain (72).

Studies on the genetics of brain tissue indicate that an interaction between tau and alpha-synuclein in (PDD) with Lewy bodies (73).

As mentioned above, specific protein kinases may hyperphosphorylate tau protein at certain sites, causing toxic isoforms of tau. Among these kinases, protein kinases A can be stimulated by alpha-synuclein, resulting in tau phosphorylation at Ser262/356 (74). Studies focused on GSK-3β, which is associated with the toxic p-tau isoform in AD, also indicate that there may be an interaction between alpha-synuclein and accumulated p-tau (75). Another study using transgenic mice that overexpressed or lacked alpha-synuclein demonstrated that alpha-synuclein is indispensable for the activation of GSK-3β. A co-IP experiment in SH-SY5Y cells also demonstrated the existence of an alpha-synuclein, p-tau, and p-GSK-3β (76) complex. Furthermore, researchers have developed a mouse model with a S9A-point mutation of human GSK-3β to investigate the relationship between alpha-synuclein and p-tau, showing a positive association between the two proteins *in vitro*, as well as in behavioral, and biochemical experiments (77, 78). Additionally, p-GSK-3β-Y216, the kinase-active form of GSK-3β, is co-localized with both p-tau and alpha-synuclein and is broadly expressed in the whole brain, while p-tau, and alpha-synuclein are expressed in TH+ DA neurons of the midbrain (78).

After showing the positive relationship between p-tau and alpha-synuclein, researchers are still investigating the mechanism underlying the toxic interaction between the two proteins. Several studies indicate that neurotoxic MPP+ induces the abnormal hyperphosphorylation of tau along with alpha-synuclein *in vitro* (79) and *in vivo* (80). Studies in a drosophila model demonstrated that tau interacting with alpha-synuclein ruined the organization of the cytoskeleton, leaving low-functioning axonal transport and structural abnormalities in neuronal synapses that resulted in PD-related cell death (81). However, the precise relationship between tau and alpha-synuclein and the molecular mechanisms responsible for PD are still unclear. There might be a cascade reaction in which the accumulation of alpha-synuclein in synapses recruits tau (82) and induces damage; the resulting low-functioning axonal transport will further promote the accumulation of tau and alpha-synuclein, and therefore, more fibrillation will be present in neurons, which will eventually lead to cell death.

## Conclusions

Tau is a key protein in many neurodegenerative diseases; however, its importance has been underestimated preoperatively in PD and PDD. Soluble, unfolded tau, after being phosphorylated or mutated, becomes insoluble and misfolded, resulting in conformational changes in microtubules and the aggregation of NFTs. The mobility of abnormal tau through brain tissue in PD is similar to prion-like diseases. The accumulation of hyperphosphorylated tau also affects axonal transport and appears to work with alpha-synuclein to contribute to tauopathy in PD and AD.

Although there is no effective treatment or drug therapy for PD and other similar neurodegenerative diseases, understanding the structure, function, and mechanism of tau and tau pathology might be helpful for early diagnosis and treatment of PD in the future.

## Author contributions

XZ and FG wrote the manuscript. DW, CL, and YF helped to edit the manuscript. JZ and WH edited the manuscript.

### Conflict of interest statement

The authors declare that the research was conducted in the absence of any commercial or financial relationships that could be construed as a potential conflict of interest.
